# When Psychiatry Meets Lipids: A case of severe hypertriglyceridemia in long-standing schizophrenia identified through a specialized metabolic clinic.

**DOI:** 10.1192/j.eurpsy.2026.11239

**Published:** 2026-07-31

**Authors:** E. Owusu, S. Ghanem, S. G. Cho, H. Shaltout, P. Chue

**Affiliations:** Psychiatry, University of Alberta, Edmonton, Canada

## Abstract

**Introduction:**

Patients with severe mental illness (SMI) treated with antipsychotics face elevated cardiometabolic risk.

**Objectives:**

We report a de-identified case of a 67-year-old woman with chronic schizophrenia and progressive negative and cognitive symptoms who developed recurrent, severe hypertriglyceridemia (HTG), culminating in a cardiovascular hospitalization.

**Methods:**

Post‑clinic plan implemented with multidisciplinary follow‑up. Serial lipid monitoring scheduled (q4–8 weeks initially).

**Results:**

Engagement with a specialized metabolic clinic led to comprehensive phenotyping, identification of a heterozygous pathogenic LPL variant (NM_000237.3:c.808C>T; p.Arg270Cys), targeted therapy, and coordinated psychiatric–metabolic care.

**Conclusions:**

Ms. A’s presentation discussed above demonstrates the crucial role genetic and pharmacologic factors play in extreme metabolic outcomes in schizophrenia. Ms. A’s case underscores the importance of integrating genomic diagnostics and metabolic expertise into psychiatric and mental health care

**Disclosure of Interest:**

None Declared

